# Correction: Trolox and Ascorbic Acid Reduce Direct and Indirect Oxidative Stress in the IPEC-J2 Cells, an *In Vitro* Model for the Porcine Gastrointestinal Tract

**DOI:** 10.1371/journal.pone.0127810

**Published:** 2015-05-01

**Authors:** The PLOS ONE Staff

## Notice of Republication

This article was republished on April 21, 2015, to correct figures that were distorted during the production process. The publisher apologizes for the error. Please download this article again to view the correct version. The originally published, uncorrected article and the republished, corrected article are provided here for reference.

## Supporting Information

S1 File Originally published, uncorrected article. (PDF)

S2 File Republished, corrected article. (PDF)
