# Supplementary material for: Correction: Trolox and Ascorbic Acid Reduce Direct and Indirect Oxidative Stress in the IPEC-J2 Cells, an In Vitro Model for the Porcine Gastrointestinal Tract
Source: PLoS One. 2015 May 1;10(5):e0127810. doi: 10.1371/journal.pone.0127810 (PMC4416923; doi:10.1371/journal.pone.0127810)
Supplement: S2 File — (PDF) [file pone.0127810.s002.pdf]

RESEARCH ARTICLE

Trolox and Ascorbic Acid Reduce Direct and Indirect Oxidative Stress in the IPEC-J2 Cells, an *In Vitro* Model for the Porcine Gastrointestinal Tract

Hans Vergauwen[1], Bart Tambuyzer[1], Karen Jennes[1], Jeroen Degroote[2,3], Wei Wang[2,3], Stefaan De Smet[3], Joris Michiels[2,3], Chris Van Ginneken[1]*

1 Laboratory of Applied Veterinary Morphology, Department of Veterinary Sciences, Faculty of Biomedical, Pharmaceutical and Veterinary Sciences, University of Antwerp, Wilrijk, Belgium, **2** Department of Applied Biosciences, Faculty of Bioscience Engineering, Ghent University, Ghent, Belgium, **3** Laboratory for Animal Nutrition and Animal Product Quality (LANUPRO), Departement of Animal Production, Faculty of Bioscience Engineering, Ghent University, Melle, Belgium

* chris.vanginneken@uantwerpen.be

Abstract

Oxidative stress in the small intestinal epithelium is a major cause of barrier malfunction and failure to regenerate. This study presents a functional *in vitro* model using the porcine small intestinal epithelial cell line IPEC-J2 to examine the effects of oxidative stress and to estimate the antioxidant and regenerative potential of Trolox, ascorbic acid and glutathione monoethyl ester. Hydrogen peroxide and diethyl maleate affected the tight junction (zona occludens-1) distribution, significantly increased intracellular oxidative stress (CM-H_2DCFDA) and decreased the monolayer integrity (transepithelial electrical resistance and FD-4 permeability), viability (neutral red) and wound healing capacity (scratch assay). Trolox (2 mM) and 1 mM ascorbic acid pre-treatment significantly reduced intracellular oxidative stress, increased wound healing capacity and reduced FD-4 permeability in oxidatively stressed IPEC-J2 cell monolayers. All antioxidant pre-treatments increased transepithelial electrical resistance and viability only in diethyl maleate-treated cells. Glutathione monoethyl ester (10 mM) pre-treatment significantly decreased intracellular oxidative stress and monolayer permeability only in diethyl maleate-treated cells. These data demonstrate that the IPEC-J2 oxidative stress model is a valuable tool to screen antioxidants before validation in piglets.

OPEN ACCESS

Citation: Vergauwen H, Tambuyzer B, Jennes K, Degroote J, Wang W, De Smet S, et al. (2015) Trolox and Ascorbic Acid Reduce Direct and Indirect Oxidative Stress in the IPEC-J2 Cells, an *In Vitro* Model for the Porcine Gastrointestinal Tract. PLoS ONE 10(3): e0120485. doi:10.1371/journal.pone.0120485

Academic Editor: Nukhet Aykin-Burns, University of Arkansas for Medical Sciences; College of Pharmacy, UNITED STATES

Received: September 4, 2014

Accepted: January 23, 2015

Published: March 6, 2015

Copyright: © 2015 Vergauwen et al. This is an open access article distributed under the terms of the Creative Commons Attribution License, which permits unrestricted use, distribution, and reproduction in any medium, provided the original author and source are credited.

Data Availability Statement: All relevant data are within the paper and its Supporting Information files.

Funding: This work was supported by the IWT_LO 100856 project provided by the agency for Innovation by Science and Technology. (http://www.iwt.be/) (CVG SDS JM). The funders had no role in study design, data collection and analysis, decision to publish, or preparation of the manuscript.

Introduction

Oxidative stress is considered one of the key players in malabsorption and inflammation of the gastrointestinal tract (GIT) as seen in necrotizing enterocolitis (NEC) [1], celiac disease [2], inflammatory bowel disease (IBD) [3] and Crohn's disease [4]. Oxidative stress has been shown to be one of the underlying pathophysiological mechanisms in a variety of diseases [5–9]. Intra

Competing Interests: The authors have declared that no competing interests exist.

uterine growth retardation (IUGR) induces oxidative stress [10] in piglets, fuelling the search for new synthetic and natural antioxidants [11–14].

The intestinal epithelium serves as an important part of the first line defence and regulate passive diffusion of solutes and macromolecules. The intestinal barrier is composed of a single layer of columnar epithelial cells sealed by tight junctions. The tight junctions can be found close to the apical side of the paracellular space. These structures are affected by oxidative stress since the pathophysiology of a redox imbalance is characterized by disrupted tight junction complexes [15–18]. Disruption of the tight junctions enables free passage of macromolecules, endotoxins or pathogens such as fluorescein sodium [19], horseradish peroxidase [20], *Escherichia coli* (strains HB101 and F18) as well as *Salmonella typhimurium* [21–23]. Next to an impaired barrier function, oxidative stress also affects mitosis and apoptosis of intestinal epithelial cells [24]. Oxidative stress distorts the normal differentiation of epithelial cells from crypt to villus, as this transition is modulated by the ratio of glutathione disulfide to reduced glutathione (GSSG/GSH) and the ratio of cysteine to cystine (Cys/CySS) [25]. Thus, maintaining a balanced redox status is crucial to ensure an optimal intestinal physiology [26].

In this study, the porcine small intestinal epithelial cell line IPEC-J2 [27], derived from the jejunum of a neonatal unsuckled piglet, was used to mimic the porcine intestinal epithelium and to examine effects of a disturbed redox state in the GIT. IPEC-J2 cells represent a suitable *in vitro* model as they produce some glycocalyx-bound mucus proteins, cytokines, chemokines and display Toll-like receptors [28–30]. Growing this non-tumorigenic, non-transformed, permanent cell line in a two chamber set-up (Boyden chamber) highly resembles the *in vivo* situation, modelling the GIT lumen and the systemic circulation [20, 30]. Furthermore, this non-tumorigenic cell line provides important insight next to a transformed cell line as they react differently to oxidative stress.

This study aimed to present a functional *in vitro* model as a useful primary tool to analyse the effects of antioxidants and feed components on membrane integrity, permeability and (non)pathogenic translocation through an epithelial monolayer exposed to oxidative stress. Oxidative stress was induced by hydrogen peroxide (H_2O_2) and diethyl maleate (DEM). Trolox, a water-soluble form of vitamin E, ascorbic acid and glutathione monoethyl ester (GSH-MEE) were used to restore the impaired redox balance. Analogous to the *in vivo* situation, the integrity of this *in vitro* epithelium depends on the viability of cells and their interconnections, i.e. the tight junctions. Therefore, the transepithelial electric resistance (TEER) was determined to assess the functional integrity of the epithelial monolayer in combination with an FITC-conjugated dextran-4 (FD-4, 4 kDa) permeability assay. Furthermore, immunocytochemical staining with zona occludens-1 (ZO-1) was performed on IPEC-J2 cells to investigate the tight junction distribution. Cell viability and proliferation were monitored using the neutral red dye. In addition, our research showed applicability of CM-H_2DCFDA in IPEC-J2 cells to investigate intracellular oxidative stress. This fluorescent probe has been previously used in different cell-based assays [31, 32]. HPLC technique was used as a direct method to determine the GSSG/GSH ratios. To our knowledge, this is the first study using the IPEC-J2 cell model to combine different modes of oxidative stress induction in relation to monolayer integrity, tight junction distribution, permeability, wound healing capacity, intracellular oxidative stress and GSSG/GSH ratio. Furthermore, 2 mM Trolox, 1 mM ascorbic acid, and 10 mM GSH-MEE were used to counteract or even fully remediate the detrimental effects of oxidative stress. Here, an integrated functional valuable tool is presented to investigate the effects of direct and indirect oxidative stress in relation to new synthetic or natural antioxidants and other feed components.

Materials and Methods

Cell line and culture conditions

The IPEC-J2 cells (ACC 701, DSMZ, Germany) were cultured in DMEM/F-12 mix (Dulbecco's modified Eagle medium, Ham's F-12 mixture), 1.5 mM HEPES, 5% (v/v) fetal bovine serum, 1% (v/v) insulin-transferrin-selenium mixture, 1% (v/v) penicillin-streptomycin mixture and 2.5 μg/mL fungizone (Invitrogen, Belgium) (37°C, 5% CO_2). The culture medium was refreshed every other day. When studying TEER and permeability, IPEC-J2 cells were seeded (1×10^5/well) at confluence in a 'Boyden chamber' insert (upper chamber, apical) on a polyethylene terephthalate (PET) membrane (1.12 cm^2, pore size 0.4 μm, ThinCert, Greiner Bio-One, Belgium) in a 12-well plate (lower chamber, basolateral, 1×10^5/well, flat bottom, Greiner Bio-One) to investigate the intracellular oxidative stress and wound healing capacity or in a 96-well plate (0.5×10^4/well, flat bottom, Greiner Bio-One) to assess viability.

Induction of oxidative stress

To induce oxidative stress, the cells were incubated with different concentrations of H_2O_2 or DEM (0–4 mM, Sigma-Aldrich, Belgium), which were added to the apical chamber for 1 hour. The stressor was removed by washing twice with DMEM/F-12 mix without additives (washing buffer). Concentrations of H_2O_2 and DEM were optimised for each specific experiment depending on the type of surface the cells were seeded upon.

Antioxidants to validate the *in vitro* model

Cells were pre-treated (overnight, 18 h) with the antioxidant Trolox (2 mM), ascorbic acid (1 mM) or GSH-MEE (10 mM) (Sigma-Aldrich) to validate the *in vitro* model and prove its functionality. The incubation time and concentrations were established based on preliminary tests and literature data [33–39]. All antioxidants were dissolved in culture medium and filtered (0.2 μm, Acrodisc Syringe Filters with HT Tuffryn Membrane, PALL, USA) before application on the IPEC-J2 cells.

Intracellular oxidative stress

The intracellular oxidative stress was analysed using a ROS-sensitive probe, 5-(and-6-)-chloromethyl-2',7'-dichlorodihydrofluorescein diacetate, acetyl ester (CM-H_2DCFDA) (Ex/Em: 492–495/517–527 nm) (Invitrogen, Belgium). The probe detects intracellular H_2O_2 and downstream products [40]. At confluence, the IPEC-J2 monolayer was pre-treated with (test group) or without (control group) antioxidants overnight. The next day, the cells were washed twice, incubated for 30 minutes with 750 μL of 8.67 μM CM-H_2DCFDA (diluted in washing buffer) after which the cells were washed twice again. Subsequently, the cells were incubated with (test group) or without (control group) oxidative stressors for 1 h, washed twice and finally the culture medium was refreshed and equilibrated for 1 h. The cells were trypsinised, and washed twice with phosphate-buffered saline solution (PBS, pH 7.4, 0.01 M) by centrifugation (450 X g, 5 min). The cells were analysed using a flow cytometer (Coulter EPICS XL-MCL, Belgium). The viability was assessed by the addition of 1 μL GelRed (VWR, Belgium) per mL cell suspension. The unstained control consisted of cells that were only incubated with culture medium to determine the autofluorescence of the cells. The stained control consisted of cells incubated solely with reactive oxygen species (ROS) detection probe to account for the ROS generated by the normal/basal energy metabolism of the cell. All analyses were performed according to flow cytometry standard procedures and for each sample at least 10,000 cells were

measured. Flow cytometric data analysis was performed using FlowJo version 10.0.6 (Treestar, Ashland, USA).

GSSG/GSH ratio

Glutathione (GSH) serves as a major endogenous antioxidant and is predominantly in the reduced state in body cells. As such, a low oxidized to reduced glutathione ratio is present. The GSH and GSSG concentrations of IPEC-J2 extracts were determined by HPLC using γ-Glu-Glu as internal standard solution, as described by Reed et al. (1980) and Yoshida (1996) [41, 42].

IPEC-J2 cells were pre-treated with or without antioxidant overnight followed by an oxidative stress treatment of 1 h. Then the cells were first deproteinised by a 10% (v/v) perchloric acid (PCA) solution before derivatization. The derivatization procedure included the reaction of 100 mM iodoacetic acid solution with thiols to form S-carboxymethyl derivatives followed by chromophore derivatization of primary amines with Sanger's reagent, i.e. 2,4-dinitrofluorobenzene (1% (v/v) in ethanol). GSH and GSSG were then separated by HPLC through an EC250/4.6 Nucleosil 120–7 NH_2 aminopropyl column (Machery-Nagel, Düren, Germany) protected by the same NH_2 guard column (CC8/4). Chromatographic runs were performed at a flow-rate of 1.5 mL/min, starting at 80% solvent A / 20% solvent B for 5 min followed by a 10 min linear gradient to 1% solvent A / 99% solvent B and a 10 min isocratic period at 1% solvent A / 99% solvent B. The column was then re-equilibrated to the initial conditions for 15 min. (solvent A: water-methanol solution (1:4, v/v), solvent B: 0.5 mol/L sodium acetate–64% methanol). The column temperature is maintained at 40°C. The UV detector was set at 365 nm for absorption measurements. GSH and GSSG were identified by retention times of authentic standards. Concentrations were determined by using the internal and external standards and expressed as μg/mL cell extract.

Viability (neutral red)

The cells were pre-treated with or without antioxidants overnight for 18 h. The cells were washed twice and incubated with or without an oxidative stressor for 1 h and washed twice again. Neutral red dye (Janssen Chimica, Belgium) was prepared as a 0.1% (w/v) stock solution and diluted 1/10 with washing buffer. After removing the culture medium, 100 μL washing buffer plus 50 μL 0.01% (w/v) neutral red dye was added to each well, and incubated for 2 h (37°C, 5% CO_2). Afterwards, the cells were washed twice with PBS. The neutral red dye is retained in lysosomes of living cells [43]. This dye is then extracted from the cells using 100 μL of a 50% (v/v) ethanol solution (in 0.05 M NaH_2PO_4). The absorbance was measured at 550 nm using a spectrophotometer (Tecan Genios).

Transepithelial electrical resistance (TEER)

When the IPEC-J2 monolayer was confluent ($\geq$ 1 kΩcm²) [44], the cells were pre-treated with or without antioxidants for 18 h. The cells were washed twice and incubated with an oxidative stressor for 1 h. The oxidative stressor was removed by washing the cells twice. The cells were incubated overnight with growth medium to achieve stable TEER values. TEER was determined using a Millicell-ERS electrical resistance system with STX01 electrodes (Millipore, Belgium) and calculated as kΩ x cm^2 by multiplying by the surface area of the monolayer (1.2 cm^2). Since TEER was quickly affected by changes in temperature, pH and washing steps, data were only collected after a stabilisation period of 1 or 2 days. The baseline resistance of a ThinCert porous membrane was approximately 50 Ωcm^2. This value was subtracted from all TEER data.

Permeability assay

When the IPEC-J2 monolayer was confluent ($\geq$ 1 kΩcm²), the cells were pre-treated with or without antioxidants overnight. The next day, the cells were washed twice and incubated with (test group) or without (control group) an oxidative stressor for 1 h. The cells were washed twice again. The permeability assay started when 500 μL culture medium containing 50 μg FD-4 (Sigma-Aldrich) was added to the apical chamber. The basolateral chamber was filled with 1.5 mL culture medium (37°C, 5% CO_2). The FD-4 was allowed to permeate overnight (18 h) from the apical to basolateral chamber. Subsequently, 100 μL of the basolateral chamber was transferred to a 96-well plate to measure the amount of permeated FD-4 using a fluorospectrophotometer (Ex/Em: 490 / 520 nm). The acquired data were processed using Magellan data analysis software (Tecan Genios, Belgium) [20].

Immunocytochemistry staining

IPEC-J2 cells grown for 2 weeks on poly-L-lysine coated glass coverslips were oxidatively stressed with 1 mM H_2O_2 or 4 mM DEM for 1 h. The cells were washed twice with phosphate-buffered saline (PBS), fixed in 4% paraformaldehyde (PFA) for 30 min at room temperature (RT), and washed again three times for 5 min in PBS enriched with 0.2% Tween-20. The cells were permeabilized for 1 h in PBS with 0.2% Tween-20, 0.2% Triton X-100 at RT, and washed three for 5 min in PBS with 0.2% Tween-20. Non-specific binding sites were blocked for 1 h at RT in PBS with 1% skim milk powder, 10% normal goat serum (NGS), 2.25% glycine (0.3 M) and 0.2% Tween-20. The cells were incubated overnight with a polyclonal rabbit anti-zona occludens-1 antibody (Santa Cruz Biotechnology, Santa Cruz, CA), diluted 1:50 in PBS with 0.2% Triton X-100, 0.2% Tween-20 and 10% NGS at 4°C, and washed again three times for 5 min in PBS with 0.2% Tween-20. Subsequently, the cells were incubated with biotinylated goat anti-rabbit antibody (Dako, Glostrup, Denmark), diluted 1:200 in PBS with 0.2% Triton X-100, 0.2% Tween-20 and 10% NGS for 1 h at RT. After three times washing for 5 min, the cells were incubated with streptavidin-horseradish peroxidase (Dako), diluted 1:200 in PBS with 0.2% Triton X-100, 0.2% Tween-20 and 10% NGS for 1 h at RT, and washed again twice for 5 min in PBS with 0.2% Tween-20 and once with double distilled water for 5 min. A positive reaction was visualized by incubating the cells with the 3,3′-diaminobenzidine chromogen (Dako) for 1–5 min at RT. The monolayers were counterstained by Carazzi's haematoxylin and cover slipped. Negative controls were obtained by omitting the primary antibody and replacing it by normal serum. Microscopic evaluation was performed using a BX 61 microscope (Olympus, Belgium) equipped with a DP 50 camera (Olympus). The surface area of the wound was measured using the analySIS-Pro software (Olympus).

Wound healing assay

Cells were seeded (1 x 10^5/well) in a 12-well plate (flat bottom, Greiner Bio-One). The cells were pre-treated with or without antioxidants overnight. The next day, the cells were washed twice and incubated with or without an oxidative stressor for 1 h. After exposure to oxidative stress, a wound was made by scratching a confluent monolayer with the tip of a 100 μL pipette. Non-adherent cells were washed off. Six hours after introduction of the wound, pictures were taken at three fixed locations of the wound using a CKX41 inverted microscope (Olympus, Belgium) equipped with a UC30 camera (Olympus). The surface area of the wound was measured using the analySIS-Pro software (Olympus).

Statistical analysis

Data are expressed as means ± standard error (SE) ($n \geq 3$), where n refers to the number of replicate experiments in time. SPSS Statistics 20 was used for statistical evaluation (SPSS software, IBM, USA). Non-parametric data were analysed using the Kruskal-Wallis test. Parametric data were analysed using a one-way analysis of variance (ANOVA) followed by post hoc tests (Bonferroni) of significance wherever the variance ratio was significant. A p value of < 0.05 was considered statistically significant.

Results

H_2O_2 and DEM increase intracellular oxidative stress

Incubating the cells with 0.5 mM H_2O_2 induced intracellular oxidative stress ($p<0.001$) (Fig. 1A). Pre-treatment with either 2 mM Trolox or 1 mM ascorbic acid prior to the 0.5 mM H_2O_2 treatment significantly reduced the intracellular oxidative stress assessed by the mean fluorescence intensity (MFI) ($p<0.001$), with no significant difference between both antioxidant-treated groups. Similarly, the 4 mM DEM-treatment increased the intracellular oxidative stress significantly ($p<0.001$) (Fig. 1B). Pre-treatment with either 2 mM Trolox ($p<0.001$), 1 mM ascorbic acid ($p<0.001$) or 10 mM GSH-MEE ($p = 0.007$), significantly reduced the MFI, with no significant difference between antioxidant-treated groups.

H_2O_2 and DEM differentially affect the GSSG/GSH ratio

Exposure to 0.5 mM H_2O_2 did not show any significant effect on the intracellular GSH concentration (data not shown), nor did it affect the GSSG/GSH ratio (Fig. 2A). DEM is used as an indirect oxidative stressor by irreversibly binding to glutathione and thereby significantly

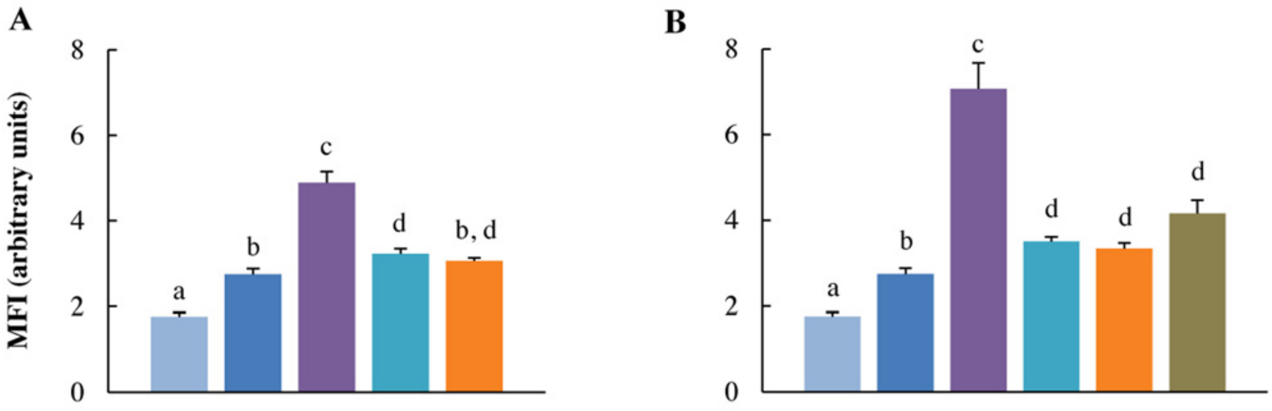

H_2O_2	-	-	+	+	+
Antioxidant	-	-	-	T	AA
Probe	-	+	+	+	+

DEM	-	-	+	+	+	+
Antioxidant	-	-	-	T	AA	G
Probe	-	+	+	+	+	+

Fig 1. H_2O_2 and DEM induce intracellular oxidative stress in IPEC-J2 cells. IPEC-J2 cells were pre-treated with or without the antioxidants Trolox, ascorbic acid or GSH-MEE and loaded with CM-H_2DCFDA probe prior to oxidative stressing. A: 0.5 mM H_2O_2, B: 4 mM DEM for 1 h. Results are presented as means ± SE, n = 3. Significant differences between treatments are represented by different letters. T: 2 mM Trolox pre-treatment, AA: 1 mM ascorbic acid pre-treatment, G: 10 mM GSH-MEE pre-treatment.

doi:10.1371/journal.pone.0120485.g001

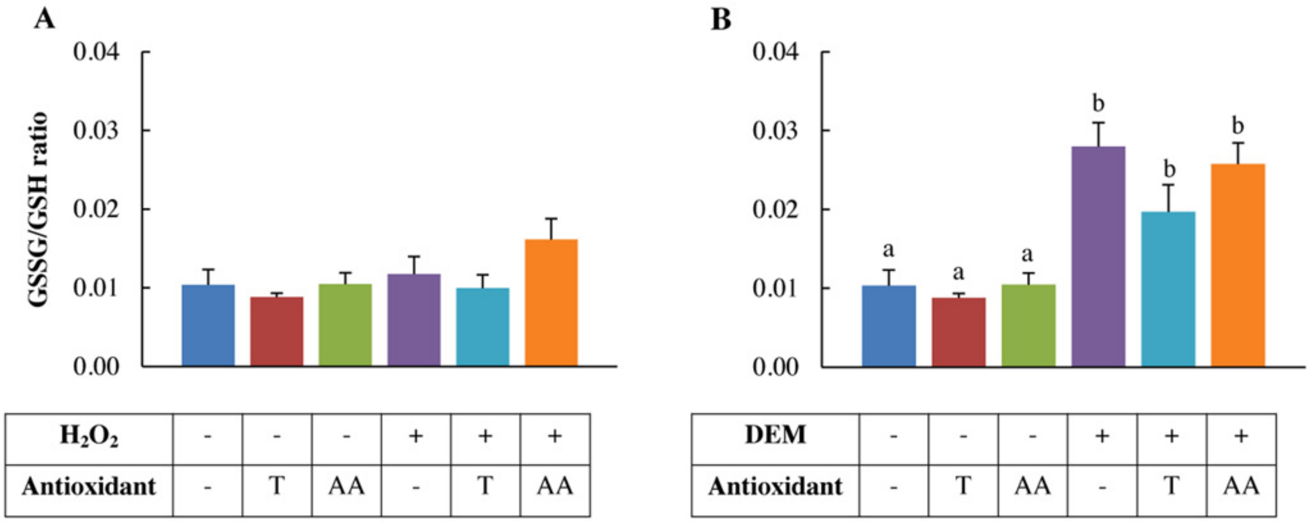

Fig 2. Effect of H_2O_2 and DEM on the intracellular oxidation state of glutathione. IPEC-J2 cells were pre-treated with or without the antioxidants Trolox or ascorbic acid and incubated with A: 0.5 mM H_2O_2, B: 4 mM DEM for 1 h. Glutathione (GSH) and glutathione disulphide (GSSG) concentrations were analysed using HPLC. Results are represented as means ± SE, n = 3. Significant differences between treatments are represented by different letters. T: 2 mM Trolox pre-treatment, AA: 1 mM ascorbic acid pre-treatment.

doi:10.1371/journal.pone.0120485.g002

decreasing the intracellular pool of glutathione (data not shown). The GSSG/GSH ratio of the 4 mM DEM-treated IPEC-J2 cells was significantly increased compared to the standard control cells ($p<0.001$). Pre-treatment using 2 mM Trolox or 1 mM ascorbic acid did not result in a significant reduction of the GSSG/GSH ratio (Fig. 2B).

H_2O_2 and DEM decrease IPEC-J2 cell viability

Exposure to 1 mM H_2O_2 ($p<0.001$) and 4 mM DEM ($p<0.001$) for 1 h resulted in acute cytotoxicity of IPEC-J2 cells (Fig. 3A-B). Trolox (2 mM) pre-treatment significantly increased the viability in 1 mM H_2O_2-treated cells ($p<0.005$) and DEM-treated cells (4 mM) ($p<0.001$) (Fig. 3A-B). 1 mM ascorbic acid pre-treatment significantly increased the viability in 4 mM DEM-treated cells ($p<0.001$) (Fig. 3B), but not in 0.5 mM H_2O_2-treated cells (Fig. 3A).

H_2O_2 and DEM decrease membrane integrity

TEER was assessed to measure the functional integrity of the epithelial monolayer. Increasing concentrations of both oxidative stressors decreased the TEER values in a concentration-dependent manner (Fig. 4A-B). Exposure to 1 mM H_2O_2 significantly reduced the TEER ($p<0.001$) and none of the antioxidant pre-treatments were able to salvage this effect (Fig. 4C). However, 2 mM Trolox pre-treatment increased numerically but not significantly the TEER compared to the 1 mM H_2O_2-stressed cells, whereas 1 mM ascorbic acid and 10 mM GSH-MEE pre-treatment did not show this trend. 10 mM GSH-MEE control had a negative effect on the monolayer integrity, as it significantly decreased the monolayer integrity compared to control values ($p = 0.021$). DEM treatment (4 mM) caused a significant reduction in TEER ($p<0.001$). However, this was rescued by all antioxidant pre-treatments resulting in higher TEER values compared to the 4 mM DEM-stressed cells ($p = 0.002$) (Fig. 4D).

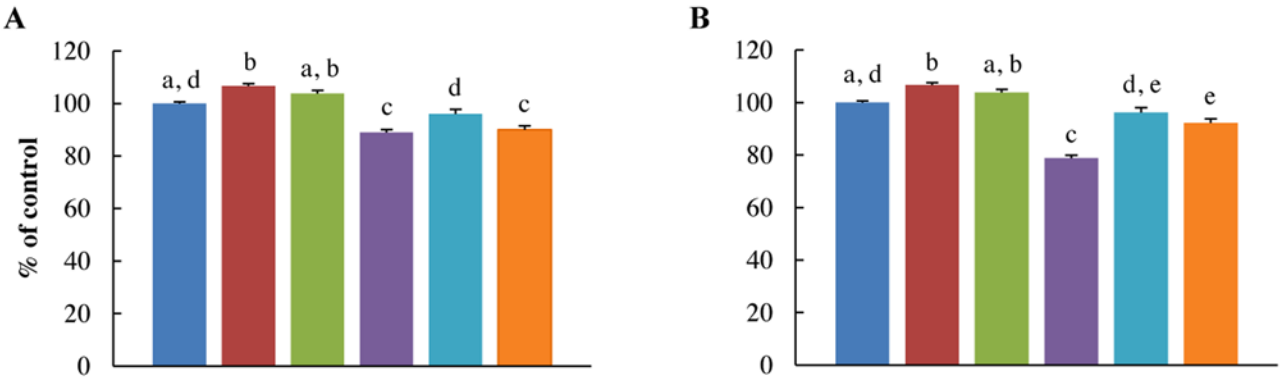

H_2O_2	-	-	-	+	+	+
Antioxidant	-	T	AA	-	T	AA

DEM	-	-	-	+	+	+
Antioxidant	-	T	AA	-	T	AA

Fig 3. Viability of H_2O_2 and DEM-treated IPEC-J2 cells pre-treated with antioxidants. IPEC-J2 cells were pre-treated with or without the antioxidants Trolox or ascorbic acid and incubated with A: 1 mM H_2O_2, B: 4 mM DEM for 1 h. Results are represented as means ± SE (n = 4), normalized to the control treatment. Significant differences between treatments are represented by different letters. T: 2 mM Trolox pre-treatment, AA: 1 mM ascorbic acid pre-treatment.

doi:10.1371/journal.pone.0120485.g003

H_2O_2 and DEM increase membrane permeability

Our data showed that the permeability of FD-4 increased depending on the concentration of both H_2O_2 and DEM (Fig. 5A-B). In 1 mM H_2O_2-stressed cells, 1 mM ascorbic acid and especially 2 mM Trolox pre-treatment resulted in a significant reduction of the amount of permeated tracer (2 mM Trolox: $p = 0.001$, 1 mM ascorbic acid: $p = 0.005$) (Fig. 5C). However, 2 mM Trolox pre-treatment was more effective than the 1 mM ascorbic acid pre-treatment ($p<0.001$). GSH-MEE pre-treatment (10 mM) was unable to significantly reduce the amount of permeated tracer. There was no significant difference between 1 mM ascorbic acid and 10 mM GSH-MEE pre-treatments. No antioxidant was able to reduce the permeability to control values ($p<0.001$). DEM-treatment (4 mM) significantly increased the FD-4 permeability compared to all other treatments ($p<0.001$) (Fig. 5D). All antioxidant pre-treatments were able to significantly reduce the membrane permeability ($p<0.001$), but none were able to reduce to control values. 2 mM Trolox pre-treatment was significantly more effective than the 1 mM ascorbic acid and 10 mM GSH-MEE pre-treatments ($p<0.001$), with no significant difference between these two pre-treatments.

H_2O_2 and DEM change the tight junction distribution

The effect of H_2O_2 and DEM on tight junction distribution was investigated by immunocytochemical staining. Zona occludens-1 (ZO-1), a tight junction-associated protein was studied in relation to oxidative stress. Microscopic illustrations show the presence of ZO-1 in the IPEC-J2 control cells that were not oxidatively stressed (Fig. 6A). Moreover, the tight junction proteins are not only present at the cell-cell contact complexes but are also diffusely present in the cell. Dividing cells show a redistribution of the tight junctions present in the cell without affecting the cell-cell contacts (Fig. 6A). However, when IPEC-J2 cells are oxidatively stressed by 1 mM H_2O_2 the IPEC-J2 cell morphology changes as vesicles appear and tight junction presence increases in the cell body (Fig. 6B). Only minor changes in cell-cell contact are detected although the distribution of the tight junction proteins in the H_2O_2-treated cells differs completely from

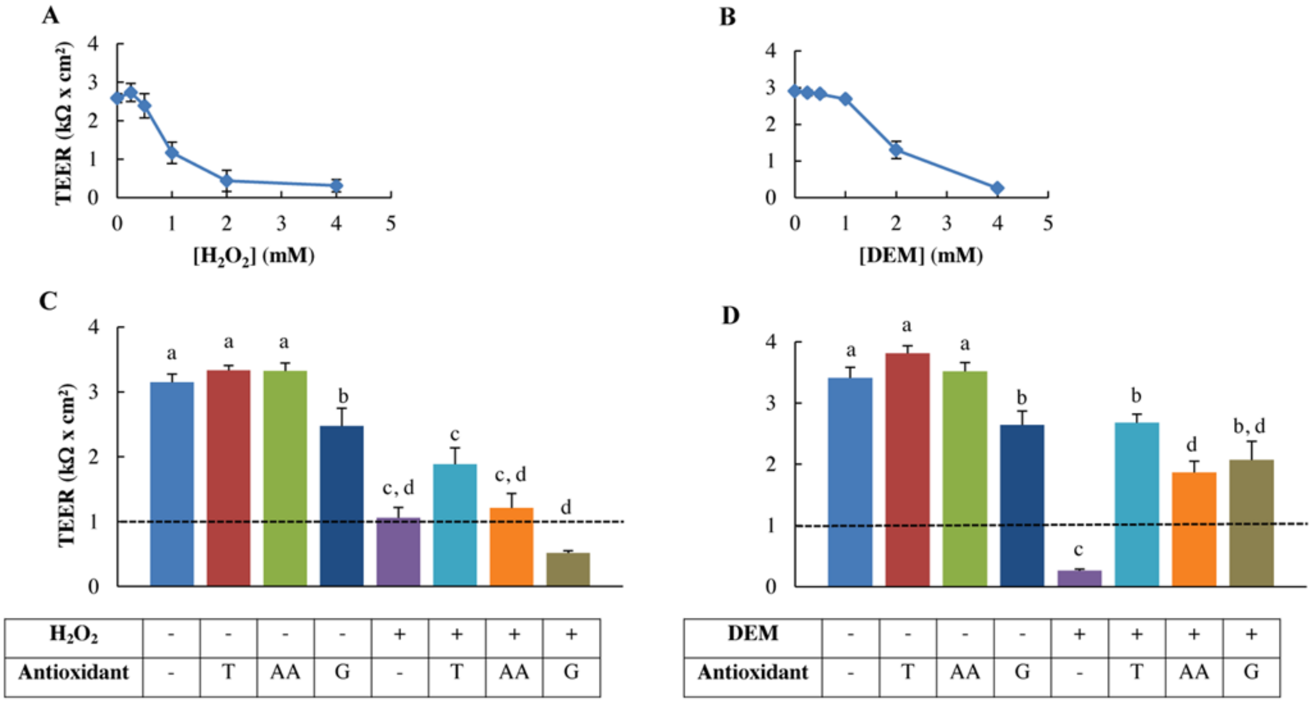

Fig 4. Membrane integrity of H_2O_2 and DEM-stressed IPEC-J2 cells with or without antioxidants pre-treatment. Membrane integrity, assessed by transepithelial electrical resistance (TEER) was determined after an overnight incubation with plain growth medium in order to stabilise the TEER values. A: TEER is significantly reduced when incubated with 1 mM H_2O_2 or more ($p<0.001$). B: TEER is significantly reduced when incubated with 2 mM DEM or more ($p<0.001$). Results are represented as means ± SE, n = 3. IPEC-J2 cells were pre-treated with or without the antioxidants Trolox, ascorbic acid or GSH-MEE and incubated with C: 1 mM H_2O_2, D: 4 mM DEM for 1 h. Results are represented as means ± SE, n = 4. The dotted black line marks the confluency threshold of 1 kΩcm². Significant differences between treatments are represented by different letters. T: 2 mM Trolox pre-treatment, AA: 1 mM ascorbic acid pre-treatment, G: 10 mM GSH-MEE pre-treatment.

doi:10.1371/journal.pone.0120485.g004

the control cells. DEM-treated IPEC-J2 cells present a large increase in loss of tight junction proteins from the cell-cell contact complexes, together with a redistribution of the tight junction proteins of the cell body (Fig. 6C).

H_2O_2 and DEM compromise the wound healing capacity

Inducing oxidative stress in IPEC-J2 cells significantly impaired wound healing capacities (1 mM H_2O_2: $p<0.001$, 4 mM DEM: $p = 0.006$) (Fig. 7A-B). Antioxidant pre-treatments significantly increased wound-healing capacities (2 mM Trolox: $p = 0.001$, 1 mM ascorbic acid: $p = 0.016$) (Fig. 7A). The impairment of the wound healing capacity of IPEC-J2 cells by oxidative stress and the beneficial effect of antioxidant pre-treatment is visualised by representative microscopic illustrations (Fig. 8). Moreover, cells treated with antioxidants prior to 0.5 mM H_2O_2 exposure regained a wound healing capacity comparable to antioxidant control cells. In 4 mM DEM-stressed cells, only 2 mM Trolox pre-treatment significantly increased the wound healing capacity ($p = 0.025$) (Fig. 7B). However, 2 mM Trolox pre-treatment was unable to restore the healing capacity to its respective control value. On the other hand, 1 mM ascorbic acid pre-treatment did not significantly increase the wound healing capacity. Finally, 2 mM Trolox showed to be more potent in promoting the regenerative capacity compared to 1 mM ascorbic acid ($p = 0.033$).

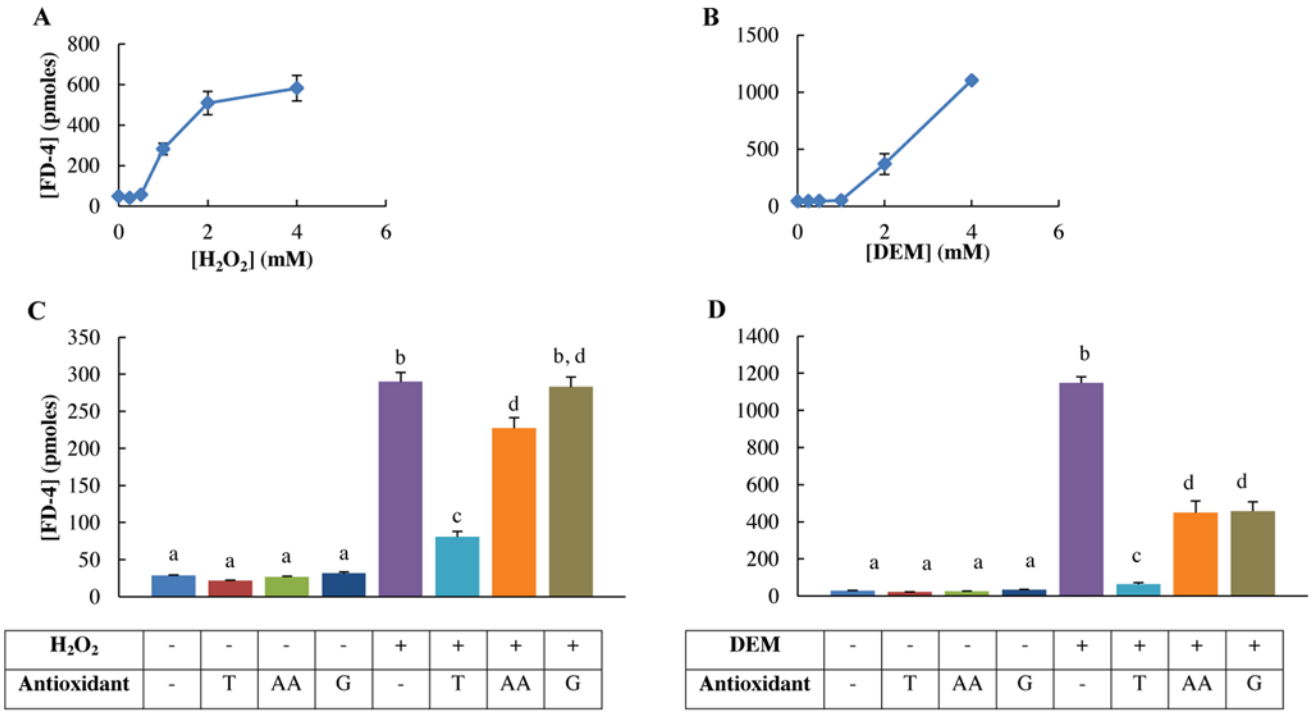

Fig 5. Membrane FD-4 permeability of H_2O_2 and DEM-stressed IPEC-J2 cells with or without antioxidant pre-treatment. Membrane permeability was quantitated one day after oxidative stressing by measuring the amount of FD-4 that permeated through the IPEC-J2 monolayer. A: FD-4 permeation is significantly increased when incubated with 1 mM H_2O_2 or more ($p<0.001$). B: FD-4 permeation is significantly increased when incubated with 2 mM DEM or more ($p<0.001$). Results are represented as means ± SE, n = 3. IPEC-J2 cells were pre-treated with or without the antioxidants Trolox, ascorbic acid or GSH-MEE and incubated with C: 1 mM H_2O_2, D: 4 mM DEM for 1 h. Results are represented as means ± SE, n = 4. Significant differences between treatments are represented by different letters. T: 2 mM Trolox pre-treatment, AA: 1 mM ascorbic acid pre-treatment, G: 10 mM GSH-MEE pre-treatment.

doi:10.1371/journal.pone.0120485.g005

Discussion

Oxidative stress impairs cellular function

ROS are generated as by-products of energy metabolism through the electron transport chain reactions in mitochondria and originate from other endogenous or exogenous sources [45]. However, oxidative stress can lead to an imbalance of the redox state impairing the cellular functionality by disrupting signalling pathways, DNA, proteins and lipid structures. Our data confirm a basal oxidative status in normal proliferating IPEC-J2 cells (stained control). This research showed that both 0.5 mM H_2O_2 and 4 mM DEM cause extensive intracellular oxidative stress in IPEC-J2 cells. To date, only H_2O_2 has been used as a direct oxidative stressor in IPEC-J2 cells [46–48], however without significantly reducing the intracellular oxidative stress by antioxidant pre-treatment [46]. The use of 0.5 or 1 mM H_2O_2 was optimized for each experiment and the chosen concentration depended on the sensitivity and timeframe of a specific experiment in order to investigate different antioxidants. In contrast to H_2O_2 which directly induces oxidative stress, DEM can be considered an indirect oxidative stressor since it irreversibly binds to glutathione, thereby depleting intracellular glutathione and diminishing the cells' oxidant scavenging capacity [49]. This is the first report of DEM being used as an indirect oxidative stress inducer in IPEC-J2 cells. It has only been used in Caco-2 cells previously [50]. Our data confirm that 4 mM DEM effectively decreased the intracellular GSH pool and significantly increased the GSSG/GSH ratio in IPEC-J2 cells.

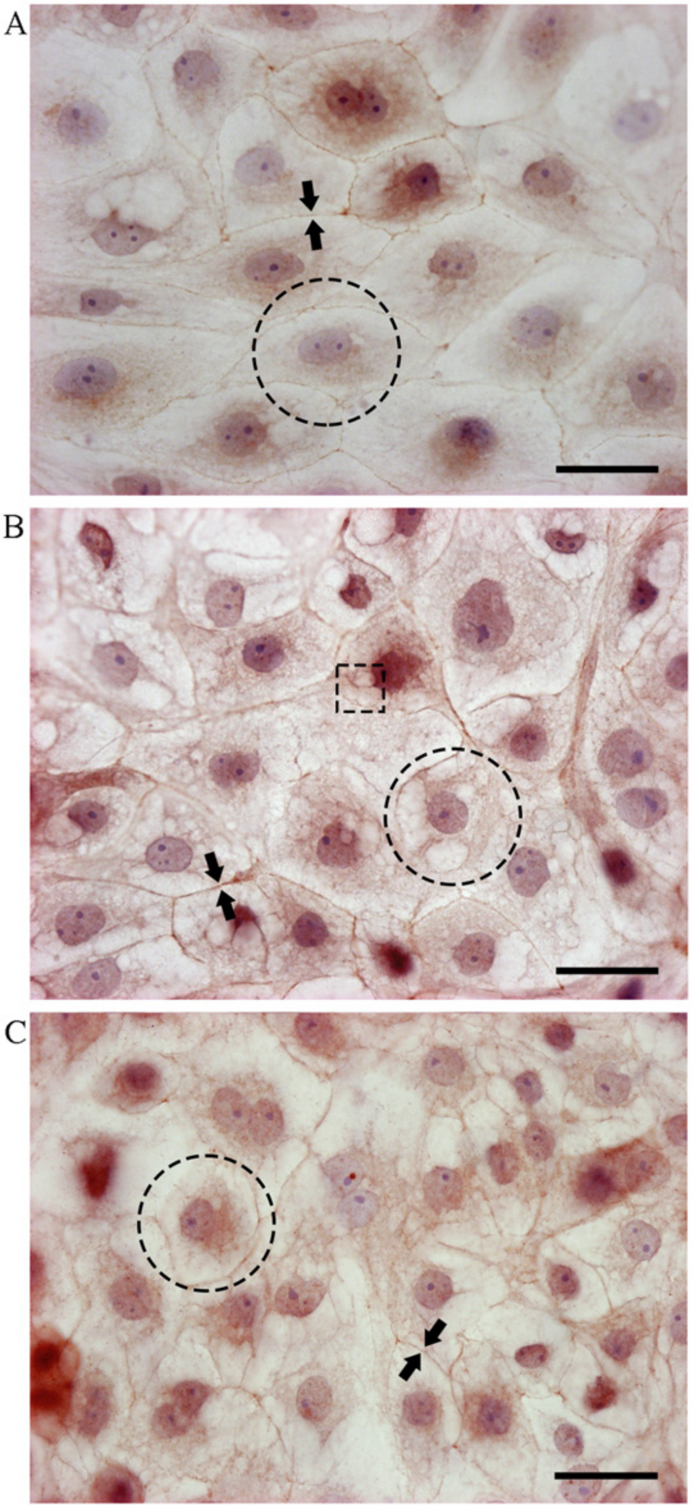

Fig 6. H_2O_2 and DEM affect the tight junction distribution in IPEC-J2 cells. Immunocytochemical staining with ZO-1 of IPEC-J2 cells was performed to investigate the effect of A: no treatment, B: 1 mM H_2O_2 or C: 4 mM DEM for 1 h on the tight junction distribution. The arrowheads point towards the tight junction-associated protein ZO-1 at the cell-cell contact complex. The dotted circle marks the tight junction distribution in an IPEC-J2 cell. The dotted square focusses on the increased presence of vesicles, especially seen in dividing or H_2O_2-stressed cells. Scale bar: 30 μm.

doi:10.1371/journal.pone.0120485.g006

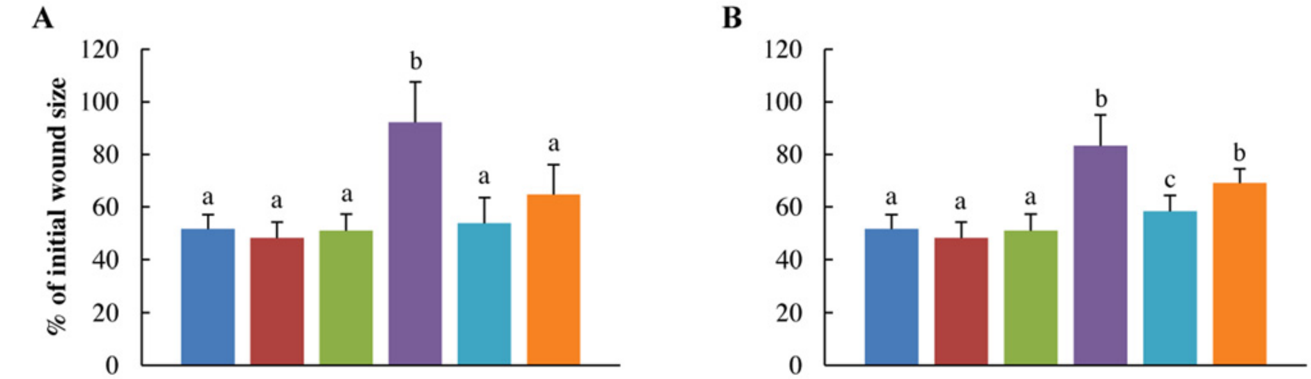

H_2O_2	-	-	-	+	+	+
Antioxidant	-	T	AA	-	T	AA

DEM	-	-	-	+	+	+
Antioxidant	-	T	AA	-	T	AA

Fig 7. Wound healing capacity of H_2O_2 and DEM-stressed IPEC-J2 cells pre-treated with antioxidants. IPEC-J2 cells were pre-treated with or without the antioxidants Trolox or ascorbic and incubated with A: 0.5 mM H_2O_2, B: 4 mM DEM for 1 h. Results are represented as means ± SE, n = 4. Significant differences between treatments are represented by different letters. T: 2 mM Trolox pre-treatment, AA: 1 mM ascorbic acid pre-treatment.

doi:10.1371/journal.pone.0120485.g007

These results also confirm that oxidative stress caused by H_2O_2 or DEM is detrimental for IPEC-J2 cell viability and the capacity to regenerate. Exposure to H_2O_2 or DEM decreased the viability of the IPEC-J2 cells in a concentration-dependent manner. Previous research using 4',6-diamidino-2-phenylindole (DAPI) staining showed that 0.5–1 mM H_2O_2 results in minimal necrosis of IPEC-J2 cells [47]. On the other hand, 3-(4, 5-dimethylthiazol-2-yl)-2, 5-diphenyltetrazolium bromide (MTT) assay showed significant decrease in IPEC-J2 cell viability when treated with 1 mM H_2O_2 [46]. Our data corroborate the latter findings, 1 mM H_2O_2 resulted in significant cell death. Regeneration capacity was significantly decreased by 0.5 mM H_2O_2 and 4 mM DEM. Intestinal epithelium can be injured by oxidative stress despite its barrier function [51]. Our findings demonstrate that an increase in oxidative stress resulted in a decreased wound healing capacity of the IPEC-J2 cells, comparable to a compromised intestinal restitution process.

TEER experiments focus on the functional integrity of a monolayer of cells and thus the stability of the tight junctions. H_2O_2 promotes tyrosine phosphorylation of different components in the tight junction complex, rendering the tight junctions unstable [15–18, 52, 53]. This results in a concentration-dependent decrease in TEER values [17, 47], which has also been demonstrated in this study. It has been demonstrated that 0.15 mM DEM can prevent H_2O_2-induced changes in TEER and mannitol flux in Caco-2 cell monolayers [54]. The latter study showed that 0.15 mM DEM did not significantly affect the TEER or mannitol flux. Our data confirm the previous study as the TEER and FD-4 permeability were only significantly affected starting from 2 mM DEM, whereas only 0.15 mM DEM was used in the previous study. Our research clearly shows a concentration-dependent effect of DEM on membrane integrity as assessed by TEER and FD-4 flux. Immunocytochemical staining of H_2O_2 and DEM-treated IPEC-J2 cells showed a redistribution of tight junction-associated protein ZO-1 and corroborate our findings that oxidative stress affects the functional integrity of the IPEC-J2 cell monolayer. The overall morphological changes in these IPEC-J2 cells are likely to be a direct result of the oxidative stress induced by 4 mM DEM or 1 Mm H_2O_2.

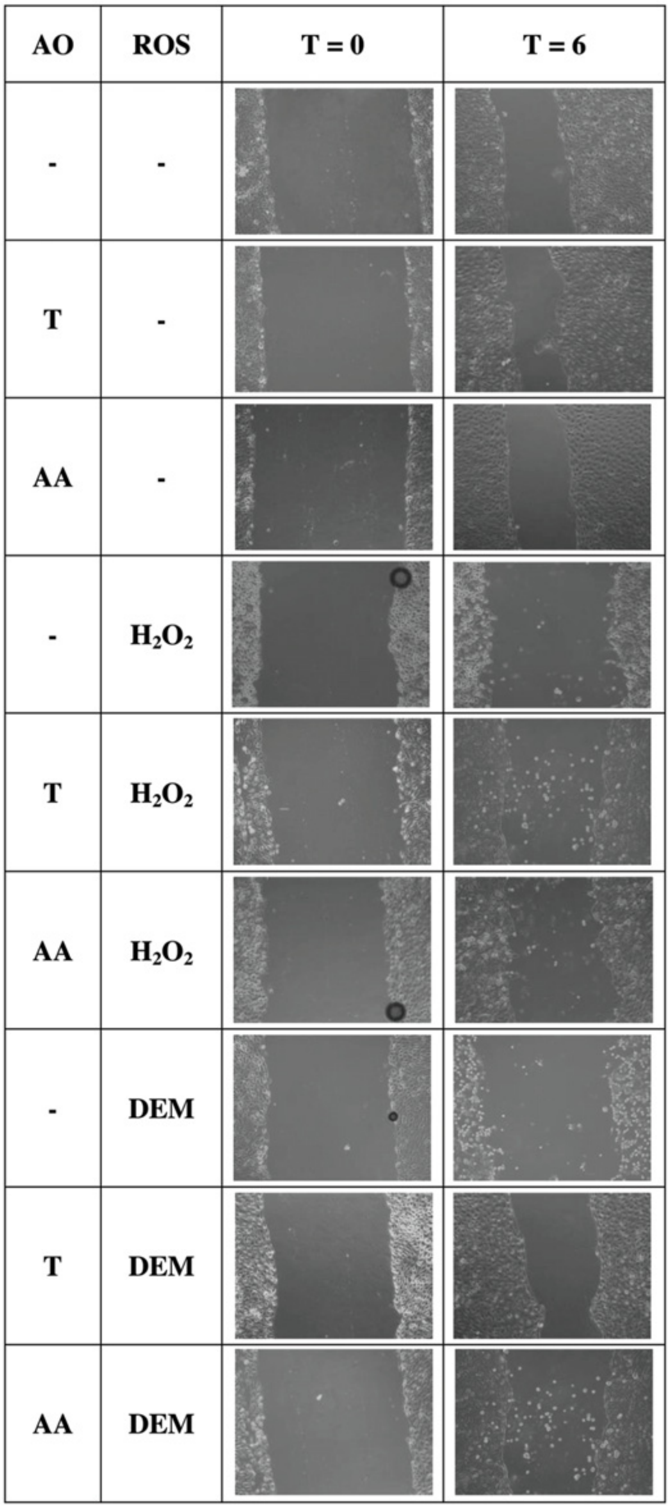

Fig 8. Microscopic representation of the wound healing capacity of H_2O_2 and DEM-stressed IPEC-J2 cells pre-treated with antioxidants. IPEC-J2 cells were pre-treated with or without the antioxidants Trolox or ascorbic and incubated with A: 0.5 mM H_2O_2, B: 4 mM DEM for 1 h. The microscopic illustrations show the start of the wound healing assay (T = 0) and the final measuring point after 6 h (T = 6). AO: antioxidant treatment, ROS: oxidative stress inducer, T: 2 mM Trolox pre-treatment, AA: 1 mM ascorbic acid pre-treatment.

doi:10.1371/journal.pone.0120485.g008

This research shows that IPEC-J2 cells are affected by H_2O_2- or DEM-induced oxidative stress in a concentration-dependent manner as determined by their viability, TEER and FD-4 permeability, regeneration capacity and intracellular oxidative stress measurement.

Reduction of direct and indirect oxidative stress in IPEC-J2 cells

IPEC-J2 cells were pre-treated overnight (18 h) with 2 mM Trolox, 1 mM ascorbic acid or 10 mM GSH-MEE to increase the cells' total antioxidative status. Prolonged exposure to antioxidants was chosen to resemble the *in vivo* situation more closely in which the intestinal mucosa and epithelial cells are exposed to nutritional antioxidants for longer periods of time during the digestive process. However, this depends on the bioaccessibility and bioavailability of a compound and the digestion time of the food matrix being studied [55].

Antioxidants can also induce oxidative stress depending on surrounding reactants and their concentrations. It has previously been demonstrated that ascorbic acid induces lipid peroxidation of biological membranes in the presence of physiologic metal ions, albeit this could be prevented by co-incubation with Trolox [56]. Therefore, specific antioxidant concentrations were investigated in the present study to avoid induction of oxidative stress by the antioxidants itself without affecting their potential to reduce the oxidative stress caused by H_2O_2 or DEM (data not shown).

Cai et al. (2013) reported that pre-treatment with lipoic acid did not significantly reduce the intracellular oxidative stress after 1 hour of 1 mM H_2O_2 treatment in IPEC-J2 cells [46]. In order to establish a functional platform to analyse new antioxidants or feed compounds, there is need for a reference antioxidant. Our data clearly indicate that both antioxidants, Trolox (2 mM) and ascorbic acid (1 mM) can significantly reduce the intracellular oxidative stress induced by 0.5–1 mM H_2O_2 or 4 mM DEM.

Incubating IPEC-J2 cells with either 1 mM H_2O_2 or 4 mM DEM caused acute cytotoxicity. This research shows that long-term antioxidant pre-incubation is an effective strategy to positively affect wound healing capacity, viability, intracellular oxidative stress, monolayer integrity (TEER and FD-4 permeation) of IPEC-J2 monolayers. These results validate this model as a powerful tool to investigate other antioxidants or feed components in relation to the basal oxidative status of the gut epithelium.

Our data show that the rescue effect of antioxidants in DEM-treated cells in general is more pronounced than in H_2O_2-treated cells. In DEM-treated cells only the function of glutathione is irreversibly blocked giving rise to an increase of oxidative stress. Antioxidant incubation can possibly counteract this effect more efficiently compared to oxidative stress caused by H_2O_2 incubation, as this stressor directly affects multiple cellular structures at once. The effect of DEM is indirect and confined to only a part of the cellular antioxidant system, namely glutathione. Incubation of different concentrations of DEM showed a "cut-off point" where the loss of functional glutathione started to affect the overall antioxidant status. In comparison, different concentrations of H_2O_2 resulted in a more gradual decline in antioxidative capacity. Therefore, the increase in antioxidative capacity by pre-incubation with 2 mM Trolox or 1 mM ascorbic acid showed to be sufficient to increase the cells' capacity to counteract the effect of the oxidative stress caused by DEM. Furthermore, the use of different doses of oxidative stressors, 4 mM DEM and 0.5–1 mM H_2O_2 could influence the potential of antioxidants to alleviate the detrimental effects of oxidative stress.

GSH-MEE pre-treatment (10 mM) has shown its potential to increase the intracellular GSH level prior to incubation with 4 mM DEM by significantly decreasing the intracellular oxidative stress and FD-4 permeability and significantly increasing the TEER. FD-4 permeability decreased and TEER increased only in 4 mM DEM-stressed cells when pre-treated with 10 mM

GSH-MEE and not in H_2O_2-treated cells. This emphasises the specificity of this compound designed to counteract DEM-mediated effects. Our data confirm previous research that elevating the intracellular GSH concentration does not increase the cells' overall ability to withstand oxidative damage by H_2O_2 [57]. Unfortunately, 10 mM GSH-MEE alone significantly decreased the TEER of the IPEC-J2 cells. Therefore, 10 mM GSH-MEE pre-treatment was excluded from the wound healing assay, GSSG/GSH assay and viability assay for both stressors, as well as from the intracellular oxidative stress test using H_2O_2. Moreover, neither 2 mM Trolox nor 1 mM ascorbic acid pre-treatment was able to prevent significant depletion of the intracellular GSH pool in 4 mM DEM-treated cells.

Our data demonstrate that 2 mM Trolox and 1 mM ascorbic acid can be used to protect the tight junction complex. Both 2 mM Trolox and 1 mM ascorbic acid pre-treatment significantly reduced FD-4 permeation in both 1 mM H_2O_2- and 4 mM DEM-stressed cells. However, 2 mM Trolox pre-treatment was significantly more effective than the 1 mM ascorbic acid pre-treatment. 2 mM Trolox and 1 mM ascorbic acid pre-treatments rescued TEER only in 4 mM DEM-treated cells. Ascorbic acid easily diffuses into the cell, while the transfer of Trolox into the cell is possibly hampered by its partial hydrophobic nature. Therefore, Trolox might be retained partially within the cellular membranes. Consequently, it will be in close proximity to the tight junction complexes where it can more easily defuse the direct oxidative effect of H_2O_2 on these protein structures. This could explain why the antioxidant effect of 2 mM Trolox is more effective than 1 mM ascorbic acid when investigating the integrity and permeability of an epithelial monolayer. On the other hand, the differential effects could also be attributed to the different antioxidant doses used or other dissimilarities in chemical properties (e.g. redox potential) between Trolox and ascorbic acid.

The regeneration capacity of IPEC-J2 cells was halted by oxidative stress, but was overcome by antioxidant pre-treatment. Both 2 mM Trolox and 1 mM ascorbic acid controls showed a significant reduction in wound size over time. IPEC-J2 cells have been previously used in a wound healing assay to elucidate the beneficial effect of sodium butyrate on diarrhoea in weanling piglets [58]. In the latter study, 4 mM sodium butyrate supplementation enhanced wound healing and exerted antioxidant properties that were significantly larger compared to 10 mM ascorbic acid when used as a control. However, 10 mM ascorbic acid promoted the upregulation of several antioxidative parameters more extensively compared to 4 mM sodium butyrate. Nonetheless, sodium butyrate could be a valid candidate to be tested in our model as it also protects HT29 tumour cells from H_2O_2-induced DNA damage [59].

Antioxidant pre-treatment with 2 mM Trolox or 1 mM ascorbic acid was able to partially prevent or even completely salvage the detrimental effects caused by H_2O_2 and DEM. This integrated model can be used as an *in vitro* tool to screen different feed additives, antioxidants or other compounds of interest prior to *in vivo* evaluation.

Acknowledgments

We thank K. Huybrechts and G. Vrolix for their technical assistance. We would like to express our gratitude to Prof. Dr. Peter Ponsaerts to grant us the use of his lab and flow cytometer.

Author Contributions

Conceived and designed the experiments: HV JD SDS JM CVG BT. Performed the experiments: HV JD WW KJ BT. Analyzed the data: HV BT. Contributed reagents/materials/analysis tools: HV SDS JM. Wrote the paper: HV SDS JM CVG BT.

References

1. Kim M, Christley S, Alverdy JC, Liu D, An G. Immature oxidative stress management as a unifying principle in the pathogenesis of necrotizing enterocolitis: insights from an agent-based model. Surgical infections. 2012; 13(1):18–32. Epub 2012/01/06. doi: 10.1089/sur.2011.057 PMID: 22217195
2. John LJ, Fromm M, Schulzke JD. Epithelial barriers in intestinal inflammation. Antioxidants & redox signaling. 2011; 15(5):1255–70. Epub 2011/02/08.
3. Rezaie A, Parker RD, Abdollahi M. Oxidative stress and pathogenesis of inflammatory bowel disease: an epiphenomenon or the cause? Digestive diseases and sciences. 2007; 52(9):2015–21. Epub 2007/04/04. PMID: 17404859
4. Ozhegov EV, Zhivotova EY, Lebedko OA, Fleishman MY, Alexeenko SA, Timoshin SS. Intensity of proliferative processes and degree of oxidative stress in the mucosa of the ileum in Crohn's disease. Bulletin of experimental biology and medicine. 2012; 152(4):420–3. Epub 2012/07/18. PMID: 22803101
5. Halliwell B. Free radicals, antioxidants, and human disease: curiosity, cause, or consequence? Lancet. 1994; 344(8924):721–4. Epub 1994/09/10. PMID: 7915779
6. Kumar A, Pant MC, Singh HS, Khandelwal S. Assessment of the redox profile and oxidative DNA damage (8-OHdG) in squamous cell carcinoma of head and neck. Journal of cancer research and therapeutics. 2012; 8(2):254–9. Epub 2012/07/31. PMID: 22842371
7. Nogueira V, Hay N. Molecular Pathways: Reactive Oxygen Species Homeostasis in Cancer Cells and Implications for Cancer Therapy. Clinical cancer research: an official journal of the American Association for Cancer Research. 2013. Epub 2013/05/31.
8. Guellich A, Damy T, Lecarpentier Y, Conti M, Claes V, Samuel JL, et al. Role of oxidative stress in cardiac dysfunction of PPARalpha-/- mice. American journal of physiology Heart and circulatory physiology. 2007; 293(1):H93–H102. Epub 2007/03/21. PMID: 17369471
9. Zafrilla P, Mulero J, Xandri JM, Santo E, Caravaca G, Morillas JM. Oxidative stress in Alzheimer patients in different stages of the disease. Current medicinal chemistry. 2006; 13(9):1075–83. Epub 2006/04/14. PMID: 16611085
10. Wang J, Chen L, Li D, Yin Y, Wang X, Li P, et al. Intrauterine growth restriction affects the proteomes of the small intestine, liver, and skeletal muscle in newborn pigs. The Journal of nutrition. 2008; 138(1):60–6. Epub 2007/12/25. PMID: 18156405
11. Kaurinovic B, Popovic M, Vlaisavljevic S, Schwartsova H, Vojinovic-Miloradov M. Antioxidant profile of Trifolium pratense L. Molecules (Basel, Switzerland). 2012; 17(9):11156–72. Epub 2012/09/20. PMID: 22990457
12. Trombino S, Cassano R, Ferrarelli T, Leta S, Puoci F, Picci N. Preparation, characterization and efficacy evaluation of synthetic biocompatible polymers linking natural antioxidants. Molecules (Basel, Switzerland). 2012; 17(11):12734–45. Epub 2012/10/30. doi: 10.3390/molecules171112734 PMID: 23103536
13. Dolara P, Bigagli E, Collins A. Antioxidant vitamins and mineral supplementation, life span expansion and cancer incidence: a critical commentary. European journal of nutrition. 2012; 51(7):769–81. Epub 2012/06/12. PMID: 22684632
14. Carrasco-Pozo C, Morales P, Gotteland M. Polyphenols Protect the Epithelial Barrier Function of Caco-2 Cells Exposed to Indomethacin through the Modulation of Occludin and Zonula Occludens-1 Expression. Journal of agricultural and food chemistry. 2013. Epub 2013/05/15.
15. Rao RK, Basuroy S, Rao VU, Karnaky KJ Jr, Gupta A. Tyrosine phosphorylation and dissociation of occludin-ZO-1 and E-cadherin-beta-catenin complexes from the cytoskeleton by oxidative stress. The Biochemical journal. 2002; 368(Pt 2):471–81. Epub 2002/08/10.
16. Rao RK, Baker RD, Baker SS, Gupta A, Holycross M. Oxidant-induced disruption of intestinal epithelial barrier function: role of protein tyrosine phosphorylation. The American journal of physiology. 1997; 273(4 Pt 1):G812–23. Epub 1997/11/14.
17. Rao R, Baker RD, Baker SS. Inhibition of oxidant-induced barrier disruption and protein tyrosine phosphorylation in Caco-2 cell monolayers by epidermal growth factor. Biochemical pharmacology. 1999; 57(6):685–95. Epub 1999/02/26. PMID: 10037455
18. Seth A, Sheth P, Rao RK. EGF Prevents Hydrogen Peroxide-Induced Tyrosine Phosphorylation and Redistribution of Tight Junction and Adherens Junction Proteins in Human Colonic Mucosa. Faseb J. 2009;23.
19. Wang N, Wang G, Hao J, Ma J, Wang Y, Jiang X, et al. Curcumin ameliorates hydrogen peroxide-induced epithelial barrier disruption by upregulating heme oxygenase-1 expression in human intestinal epithelial cells. Digestive diseases and sciences. 2012; 57(7):1792–801. Epub 2012/03/07. doi: 10.1007/s10620-012-2094-7 PMID: 22392462

20. Geens MM, Niewold TA. Optimizing culture conditions of a porcine epithelial cell line IPEC-J2 through a histological and physiological characterization. Cytotechnology. 2011; 63(4):415–23. Epub 2011/06/01. doi: 10.1007/s10616-011-9362-9 PMID: 21626283

21. Yu LC, Wang JT, Wei SC, Ni YH. Host-microbial interactions and regulation of intestinal epithelial barrier function: From physiology to pathology. World journal of gastrointestinal pathophysiology. 2012; 3(1):27–43. Epub 2012/03/01. doi: 10.4291/wjgp.v3.i1.27 PMID: 22368784

22. McCracken VJ, Lorenz RG. The gastrointestinal ecosystem: a precarious alliance among epithelium, immunity and microbiota. Cellular microbiology. 2001; 3(1):1–11. Epub 2001/02/24. PMID: 11207615

23. Lewis K, Lutgendorff F, Phan V, Soderholm JD, Sherman PM, McKay DM. Enhanced translocation of bacteria across metabolically stressed epithelia is reduced by butyrate. Inflammatory bowel diseases. 2010; 16(7):1138–48. Epub 2009/12/22. doi: 10.1002/ibd.21177 PMID: 20024905

24. Herring TA, Cuppett SL, Zempleni J. Genomic implications of H(2)O (2) for cell proliferation and growth of Caco-2 cells. Digestive diseases and sciences. 2007; 52(11):3005–15. Epub 2007/06/29. PMID: 17597414

25. Circu ML, Aw TY. Intestinal redox biology and oxidative stress. Seminars in cell & developmental biology. 2012; 23(7):729–37. Epub 2012/04/10.

26. Blikslager AT, Moeser AJ, Gookin JL, Jones SL, Odle J. Restoration of barrier function in injured intestinal mucosa. Physiol Rev. 2007; 87(2):545–64. Epub 2007/04/13. PMID: 17429041

27. Berschneider HM. Development of normal cultured small intestinal epithelial cell lines which transport Na and Cl. Gastroenterology. 1989; 96:A41.

28. Mariani V, Palermo S, Fiorentini S, Lanubile A, Giuffra E. Gene expression study of two widely used pig intestinal epithelial cell lines: IPEC-J2 and IPI-2I. Veterinary immunology and immunopathology. 2009; 131(3–4):278–84. Epub 2009/05/19. doi: 10.1016/j.vetimm.2009.04.010 PMID: 19477023

29. Brosnahan AJ, Brown DR. Porcine IPEC-J2 intestinal epithelial cells in microbiological investigations. Veterinary microbiology. 2012; 156(3–4):229–37. Epub 2011/11/15. doi: 10.1016/j.vetmic.2011.11.010 PMID: 22221382

30. Schierack P, Nordhoff M, Pollmann M, Weyrauch KD, Amasheh S, Lodemann U, et al. Characterization of a porcine intestinal epithelial cell line for in vitro studies of microbial pathogenesis in swine. Histochemistry and cell biology. 2006; 125(3):293–305. Epub 2005/10/11. PMID: 16215741

31. Testa MP, Alvarado O, Wournell A, Lee J, Guilford FT, Henriksen SH, et al. Screening assay for oxidative stress in a feline astrocyte cell line, G355–5. Journal of visualized experiments: JoVE. 2011(53): e2841. Epub 2011/07/22. doi: 10.3791/2841 PMID: 21775965

32. Halliwell B, Whiteman M. Measuring reactive species and oxidative damage in vivo and in cell culture: how should you do it and what do the results mean? British journal of pharmacology. 2004; 142(2): 231–55. Epub 2004/05/25. PMID: 15155533

33. Dymkowska D, Wojtczak L. Arachidonic acid-induced apoptosis in rat hepatoma AS-30D cells is mediated by reactive oxygen species. Acta biochimica Polonica. 2009; 56(4):711–5. Epub 2009/12/02. PMID: 19949744

34. Bizzozero OA, Reyes S, Ziegler J, Smerjac S. Lipid peroxidation scavengers prevent the carbonylation of cytoskeletal brain proteins induced by glutathione depletion. Neurochemical research. 2007; 32(12):2114–22. Epub 2007/06/07. PMID: 17551832

35. Fagundes DS, Gonzalo S, Grasa L, Castro M, Arruebo MP, Plaza MA, et al. Trolox reduces the effect of ethanol on acetylcholine-induced contractions and oxidative stress in the isolated rabbit duodenum. Revista espanola de enfermedades digestivas: organo oficial de la Sociedad Espanola de Patologia Digestiva. 2011; 103(8):396–401. Epub 2011/08/27.

36. Samarasinghe DA, Tapner M, Farrell GC. Role of oxidative stress in hypoxia-reoxygenation injury to cultured rat hepatic sinusoidal endothelial cells. Hepatology. 2000; 31(1):160–5. Epub 1999/12/29. PMID: 10613741

37. Tupe RS, Agte VV. Effect of water soluble vitamins on Zn transport of Caco-2 cells and their implications under oxidative stress conditions. European journal of nutrition. 2010; 49(1):53–61. Epub 2009/08/15. doi: 10.1007/s00394-009-0048-4 PMID: 19680705

38. Kelly-Aubert M, Trudel S, Fritsch J, Nguyen-Khoa T, Baudouin-Legros M, Moriceau S, et al. GSH monoethyl ester rescues mitochondrial defects in cystic fibrosis models. Human molecular genetics. 2011; 20(14):2745–59. Epub 2011/04/27. doi: 10.1093/hmg/ddr173 PMID: 21518732

39. Zeevalk GD, Manzino L, Sonsalla PK, Bernard LP. Characterization of intracellular elevation of glutathione (GSH) with glutathione monoethyl ester and GSH in brain and neuronal cultures: relevance to Parkinson's disease. Experimental neurology. 2007; 203(2):512–20. Epub 2006/10/20. PMID: 17049515

40. LeBel CP, Ischiropoulos H, Bondy SC. Evaluation of the probe 2',7'-dichlorofluorescin as an indicator of reactive oxygen species formation and oxidative stress. Chemical research in toxicology. 1992; 5(2):227–31. Epub 1992/03/01. PMID: 1322737

41. Reed DJ, Babson JR, Beatty PW, Brodie AE, Ellis WW, Potter DW. High-performance liquid chromatography analysis of nanomole levels of glutathione, glutathione disulfide, and related thiols and disulfides. Analytical biochemistry. 1980; 106(1):55–62. Epub 1980/07/15. PMID: 7416469

42. Yoshida T. Determination of reduced and oxidized glutathione in erythrocytes by high-performance liquid chromatography with ultraviolet absorbance detection. Journal of chromatography B, Biomedical applications. 1996; 678(2):157–64. Epub 1996/04/12. PMID: 8738017

43. Repetto G, del Peso A, Zurita JL. Neutral red uptake assay for the estimation of cell viability/cytotoxicity. Nature protocols. 2008; 3(7):1125–31. Epub 2008/07/05. doi: 10.1038/nprot.2008.75 PMID: 18600217

44. Diesing AK, Nossol C, Danicke S, Walk N, Post A, Kahlert S, et al. Vulnerability of polarised intestinal porcine epithelial cells to mycotoxin deoxynivalenol depends on the route of application. PloS one. 2011; 6(2):e17472. Epub 2011/03/03. doi: 10.1371/journal.pone.0017472 PMID: 21364771

45. Brieger K, Schiavone S, Miller FJ Jr., Krause KH. Reactive oxygen species: from health to disease. Swiss medical weekly. 2012; 142:w13659. Epub 2012/08/21. doi: 10.4414/smw.2012.13659 PMID: 22903797

46. Cai X, Chen X, Wang X, Xu C, Guo Q, Zhu L, et al. Pre-protective effect of lipoic acid on injury induced by H(2)O (2) in IPEC-J2 cells. Molecular and cellular biochemistry. 2013. Epub 2013/02/27.

47. Paszti-Gere E, Csibrik-Nemeth E, Szeker K, Csizinszky R, Jakab C, Galfi P. Acute oxidative stress affects IL-8 and TNF-alpha expression in IPEC-J2 porcine epithelial cells. Inflammation. 2012; 35(3):994–1004. Epub 2011/11/16. doi: 10.1007/s10753-011-9403-8 PMID: 22083491

48. Paszti-Gere E, Csibrik-Nemeth E, Szeker K, Csizinszky R, Palocz O, Farkas O, et al. Lactobacillus plantarum 2142 prevents intestinal oxidative stress in optimized in vitro systems. Acta physiologica Hungarica. 2013; 100(1):89–98. Epub 2013/03/09. doi: 10.1556/APhysiol.100.2013.1.9 PMID: 23471044

49. Boyland E, Chasseaud LF. Enzyme-catalysed conjugations of glutathione with unsaturated compounds. The Biochemical journal. 1967; 104(1):95–102. Epub 1967/07/01. PMID: 6035529

50. Ebert B, Seidel A, Lampen A. Phytochemicals induce breast cancer resistance protein in Caco-2 cells and enhance the transport of benzo[a]pyrene-3-sulfate. Toxicological sciences: an official journal of the Society of Toxicology. 2007; 96(2):227–36. Epub 2006/11/02.

51. Banan A, Choudhary S, Zhang Y, Fields JZ, Keshavarzian A. Oxidant-induced intestinal barrier disruption and its prevention by growth factors in a human colonic cell line: role of the microtubule cytoskeleton. Free radical biology & medicine. 2000; 28(5):727–38. Epub 2000/04/08.

52. Staddon JM, Herrenknecht K, Smales C, Rubin LL. Evidence that tyrosine phosphorylation may increase tight junction permeability. Journal of cell science. 1995; 108 (Pt 2):609–19. Epub 1995/02/01.

53. Kale G, Naren AP, Sheth P, Rao RK. Tyrosine phosphorylation of occludin attenuates its interactions with ZO-1, ZO-2, and ZO-3. Biochemical and biophysical research communications. 2003; 302(2): 324–9. Epub 2003/02/27. PMID: 12604349

54. Rao RK, Li L, Baker RD, Baker SS, Gupta A. Glutathione oxidation and PTPase inhibition by hydrogen peroxide in Caco-2 cell monolayer. American journal of physiology Gastrointestinal and liver physiology. 2000; 279(2):G332–40. Epub 2000/08/01. PMID: 10915642

55. Palafox-Carlos H, Ayala-Zavala JF, Gonzalez-Aguilar GA. The role of dietary fiber in the bioaccessibility and bioavailability of fruit and vegetable antioxidants. Journal of food science. 2011; 76(1):R6–R15. Epub 2011/05/04. doi: 10.1111/j.1750-3841.2010.01957.x PMID: 21535705

56. Britt SG, Chiu VW, Redpath GT, VandenBerg SR. Elimination of ascorbic acid-induced membrane lipid peroxidation and serotonin receptor loss by Trolox-C, a water soluble analogue of vitamin E. Journal of receptor research. 1992; 12(2):181–200. Epub 1992/01/01. PMID: 1583621

57. Spector A, Huang RR, Wang GM, Schmidt C, Yan GZ, Chifflet S. Does elevated glutathione protect the cell from H2O2 insult? Experimental eye research. 1987; 45(3):453–65. Epub 1987/09/01. PMID: 3666067

58. Ma X, P XF, Li LS, Qiao SY, Zhang GL, Li DF. Butyrate promotes the recovering of intestinal wound healing through its positive effect on the tight junctions. Journal of animal science. 2012; 90 Suppl 4:266–8. Epub 2013/02/13. doi: 10.2527/jas.50965 PMID: 23365351

59. Rosignoli P, Fabiani R, De Bartolomeo A, Spinozzi F, Agea E, Pelli MA, et al. Protective activity of butyrate on hydrogen peroxide-induced DNA damage in isolated human colonocytes and HT29 tumour cells. Carcinogenesis. 2001; 22(10):1675–80. Epub 2001/09/29. PMID: 11577008
